# Dairy sheep production research at the University of Wisconsin-Madison, USA – a review

**DOI:** 10.1186/2049-1891-5-22

**Published:** 2014-04-16

**Authors:** David L Thomas, Yves M Berger, Brett C McKusick, Claire M Mikolayunas

**Affiliations:** 1 University of Wisconsin-Madison, Department of Animal Sciences, Madison, Wisconsin, USA; 2 Spooner Agricultural Research Station, Spooner, Wisconsin, USA; 3 Current address: Merial Ltd, Duluth, Georgia, USA; 4 Current address: Midwest Organic Services Association, Viroqua, Wisconsin, USA

**Keywords:** Dairy sheep, East Friesian, Grazing, Lacaune, Lactation physiology, Nitrogen efficiency, RDP, RUP, Supplementation

## Abstract

Commercial milking of sheep is a new agricultural industry in the United States starting approximately 30 yr ago. The industry is still small, but it is growing. The majority of the sheep milk is used in the production of specialty cheeses. The United States is the major importer of sheep milk cheeses with 50 to 60% of annual world exports coming to the United States during the past 20 yr. Therefore, there is considerable growth potential for the industry in the United States. The only dairy sheep research flock in North America is located at the Spooner Agricultural Research Station of the University of Wisconsin-Madison. The research program started in 1993 and has been multifaceted; dealing with several areas important to commercial dairy sheep farmers. The East Friesian and Lacaune dairy breeds were compared and introduced to the industry through the research program. Both dairy breeds produced significantly more milk than traditional meat-wool breeds found in the U.S., but the two breeds differed in their production traits. East Friesian-cross ewes produced more lambs and slightly more milk than Lacaune-cross ewes whereas Lacaune-cross ewes produced milk with a higher percentage of fat and protein than East Friesian-cross ewes. Lactation physiology studies have shown that ewes with active corpora lutea have increased milk yields, oxytocin release during milking is required to obtain normal fat percentages in the milk, large udder cisterns of dairy ewes can allow for increased milking intervals, and short daylengths during late pregnancy results in increased milk yield. In the nutrition area, legume-grass pastures and forages with a higher percentage of legume will result in increased milk production. Grazing ewes respond to additional supplementation with increased milk yield, but it is important to match the supplement to the quality of the grazing. Ewes on high quality legume-grass pastures that are high in rumen degradable protein respond with increased milk production to supplements high in energy and/or high in rumen undegraded protein.

## Brief history of the Spooner Agricultural Research Station

The Spooner Agricultural Research Station is the site of the only dairy sheep research flock in North America. The station is located in northwestern Wisconsin (N 45.8, W 91.9) and is the oldest of the 12 research stations operated throughout Wisconsin by the College of Agricultural and Life Sciences of the University of Wisconsin-Madison. The station was established in 1909 with a donation of 32 hectares of land to the University of Wisconsin by the city of Spooner. The station currently occupies 157 hectares and has active research programs in field crops, pasture, horticulture, and sheep production. The environment is temperate, exhibiting four distinct seasons during the year. The average monthly high temperature occurs in July (27°C) and the average low monthly temperature occurs in January (-17°C). Average annual precipitation (rain or snow) is 76 cm and it occurs throughout the year with the greatest amount in August (106 mm) and the least amount in January (18 mm).

Sheep were added to the station in 1936 after an outbreak of brucellosis in the small dairy cow herd resulted in disposal of the cattle. The original ewes were commercial western whiteface, and these were graded-up to Shropshire. With changes in breed popularity and needs for research, the flock over time was composed of Shropshire, Suffolk, Targhee, Finnsheep-Targhee, Romanov-Targhee, Dorset-cross, and the present dairy breeding of East Friesian (EF) and Lacaune (LA).

The first dairy sheep research program in North America was established by Dr. William Boylan at the University of Minnesota in 1984 [1], but this program ceased in about 1996 with his retirement. The dairy sheep program at the Spooner Station had its start in the summer of 1993 with the importation of two ½ EF rams from the flock of Hani Gasser, Chase, British Columbia, Canada. Construction on the sheep milking parlor started in April 1995. The milking system is a double-12, high-line, Casse system with a pit for the milkers (DeLaval®). The initial parlor had 6 milking units requiring manual cleaning. These original milking units were increased to 12 with in-place cleaning of machines. The first ewes were milked starting in April 1996, and with the collection of the first milk yield data shortly after, the dairy sheep research program began. From the years just prior to the introduction of dairy sheep through to the present time, the ewe flock has been maintained at approximately 300 breeding ewes. At the present time, the program at the Spooner Station is the only dairy sheep research program in North America.

### Dairy sheep production research

Summaries of the results of some studies conducted at the station on dairy sheep production follow. The studies selected for summarization are those whose results may have the greatest opportunity for the improvement of the efficiency of dairy sheep production. In addition, several studies have been conducted in the Department of Food Science and the Center for Dairy Research, University of Wisconsin-Madison on various processing aspects of sheep milk, and these are not summarized here. Results of most of the processing studies can be found in the Journal of Dairy Science by searching for the main authors of W. Wendorff and J. Jaeggi.

The successful completion of these studies was due in large part to the dedicated staff at the Spooner Agricultural Research Station. We (D. Thomas and Y. Berger) also had the opportunity during the past several yr to work with two excellent graduate students who are co-authors of this paper, Brett McKusick and Claire Mikolayunas, who earned their M.S. and Ph.D. degrees in Animal Sciences through these projects. They not only conducted the work, but also conceived the hypotheses and designed the experiments for most of the following studies. Without their efforts, there would be much less to present in this review.

### Breed comparisons

#### Low percentage East Friesian-crosses compared to Dorset-crosses

The first dairy sheep genetics available in North America for commercial dairy sheep production were on the farm of Hani Gasser, Chase, British Columbia, Canada who had imported frozen semen of EF rams from Switzerland and used it to inseminate his Rideau ewes. The Rideau is a composite breed developed by the Centre for Food and Animal Research (formerly Animal Research Institute), at Ottawa, Ontario, Canada [2]. The Rideau breed is estimated to be 40% Finnsheep, 20% Suffolk, 14% EF, 9% Shropshire, and 8% Dorset breeding with less than 1% additional contribution from each of the following breeds: North Country Cheviot, Leicester, Romnelet, and Corriedale. From Mr. Gasser, we purchased two 1/2 EF × 1/2 Rideau rams in 1993, one 3/4 EF × 1/4 Rideau ram in 1994, and one 7/8 EF × 1/8 Rideau ram in 1995. Three different Swiss EF rams sired the four EF-cross rams that we purchased.

Crossbred ewes of 1/2 Dorset × 1/4 (Romanov or Finnsheep) × 1/4 Targhee breeding (commercial ewes) were randomly assigned to either an EF-cross ram or polled Dorset ram in a single-sire mating pen during the late summers or autumns of the four years from 1993 to 1996. The Dorset rams were purchased from Wisconsin breeders from rams consigned to the Wisconsin Ram Test Station. Most female lambs resulting from these matings were retained as replacements and mated to either Dorset or EF-cross rams. The mating system resulted in the production of EF-cross lambs and ewes of 12.5 to 50% EF breeding and Dorset-cross lambs and ewes of 75 or 87.5% Dorset breeding and 0.0% EF breeding.

Growth data were available for 420 lambs from EF-cross sires and 216 lambs from Dorset sires on commercial ewes and an additional 546 lambs from EF-cross dams and 150 lambs from Dorset-cross dams. Reproduction data were collected from 338 matings of EF-cross ewes and 146 matings of Dorset-cross ewes. Milk production data was reported from 246 EF-cross lactations and 76 Dorset-cross lactations collected in 1996 and 1997 from one- and two-year-old ewes [3].

East Friesian-cross lambs had greater (*P* < 0.05) birth, weaning, and postweaning weights than Dorset-cross lambs. When lambing at 1 and 2 yr of age, EF-cross ewes gave birth to 0.27 more (*P* < 0.05) lambs per ewe lambing, reared 0.15 more (*P* < 0.05) lambs per ewe mated, had 33.5 more (*P* < 0.05) d in lactation, and produced 1.9 times more (*P* < 0.05) milk and more (*P* < 0.05) weight of milk fat (+2.2 kg) and milk protein (+2.2 kg) than Dorset-cross ewes (Table 1). The EF-cross ewes produced milk with a lower (*P* < 0.05) percentage fat and protein compared to Dorset-cross ewes which was expected given the negative phenotypic and genetic correlations between milk yield and percentage milk fat and protein that have been reported by several authors (e.g., [4]). The EF-cross ewes and lambs in this study were of 12.5 to 50.0% EF breeding and provided a strong endorsement for the use of dairy sheep genetics over domestic meat/wool genetics for commercial sheep dairies in North America.

**Table 1:** **Lactation performance**^**1 **^**of young East Friesian**-**cross and Dorset**-**cross ewes** (**1996 and 1997**)

**Trait**	**Breeding of ewe**	
	**Dorset**-**cross**^**3**^	**East Friesian**-**cross**^**2**^
Number of lactations	76	246
Lactation length, d	92.7^a^	126.2^b^
Milk yield, kg	56.9^a^	109.1^b^
Fat,%	5.5^a^	5.0^b^
Fat yield, kg	3.3^a^	5.5^b^
Protein,%	5.4^a^	5.0^b^
Protein yield, kg	3.2^a^	5.4^b^
Somatic cell count, log_10_	4.99	5.02

^1^Ewes were milked starting approximately 30 d after parturition.

^2^75% or 87.5% Dorset breeding and 0.0% East Friesian breeding.

^3^12.5% to 50% East Friesian breeding.

^a,b^Means within a row with no superscript in common are different (*P* < 0.05).

#### Crossbred performance of East Friesian and Lacaune breeds

As purebred rams and semen of the EF breed became available after 1995, 14 different EF rams were used in the flock. In addition, the first LA genetics in the U.S. was imported by the Spooner Station from the U.K. (semen from 3 rams) and Canada (3 rams) in 1998. Since very few, if any, additional EF and LA rams or semen from EF rams have been imported into the U.S. beyond these 14 EF and 6 LA rams, the Spooner flock is well-representative of the EF and LA genetics currently present in the U.S.

An analysis of growth, reproduction, and milk production data collected from 1999 through 2004 from lambs and ewes born between 1999 and 2004 and sired by either purebred EF or LA rams were reported [5]. Records were available on 1,749 lambs for growth, 942 exposures of 483 ewes for reproduction, and 796 lactations from 402 ewes. Lambs sired by EF rams had greater (*P* < 0.05) 30-d weights than lambs sired by LA rams but there were no differences between sire breeds for birth or 150-d weights (Table 2). Ewes sired by EF rams gave birth to 0.16 more (*P* < 0.05) lambs per ewe lambing than did ewes sired by LA rams (1.85 vs. 1.69, respectively) (Table 2). Ewes sired by EF rams produced 14.6 kg more (not statistically significant) milk per lactation than did ewes sired by LA rams. However, the greater (*P* < 0.05) percentage of both fat and protein of milk produced by LA-sired ewes compared to EF-sired ewes resulted in very similar amounts of fat and protein produced in a lactation by ewes sired by the two breeds.

**Table 2:** **Performance of lambs and ewes sired by purebred East Friesian** (**n** = **14**) **or purebred Lacaune** (**n** = **6**) **rams**^**1**^

**Trait**	**Breed of sire of lamb or ewe**	
	**East Friesian**	**Lacaune**
Lamb growth: (n = 1,794 lambs born)		
Birth wt., kg	5.04 ± 0.09^a^	4.64 ± 0.09^b^
30-d wt., kg	14.3 ± 0.2^a^	13.3 ± 0.3^b^
150-d wt., kg	48.4 ± 1.1^a^	48.9 ± 1.2^a^
Ewe reproduction: (n = 942 exposures)		
Fertility (ewes lambing/ewes exposed),%	96.7 ± 1.4^a^	94.6 ± 1.4^a^
Prolificacy (lambs born/ewes lambing), no.	1.85 ± 0.06^a^	1.69 ± 0.07^b^
Ewe lactation: (n = 796 lactations)		
Lactation length, d	161.4 ± 3.8^a^	155.2 ± 4.0^a^
Milk yield, kg	209.4 ± 9.8^a^	194.8 ± 11.5^a^
Fat yield, kg	12.3 ± 0.6^a^	12.5 ± 0.7^b^
Fat,%	5.75 ± 0.09^b^	6.31 ± 0.11^a^
Protein yield, kg	10.3 ± 0.5^a^	10.1 ± 0.6^b^
Protein,%	4.81 ± 0.06^b^	5.15 ± 0.06^a^

^1^Performance data were collected from 1999 through 2004 from lambs and ewes born between 1999 and 2004.

^a,b^Means within a row with no superscript in common are different (*P* < 0.05).

A more sophisticated analysis on all records collected from 1996 through 2005 was conducted by J. Casellas (unpublished), which took into consideration the effects of ewe breed composition, proportion of retained heterosis, weaning system, age of ewe, and number of lambs born on lactation traits and the same effects, except number of lambs born, for the trait of litter size. Performance data were available on 1,068 ewes with 2,554 lactation records. Using the regression of ewe performance for each trait on proportion of EF or LA breeding, the predicted performance of purebred EF and LA ewes was estimated. Presented in Table 3 is the predicted performance of purebred EF and LA ewes at 3 yr of age when they are milked starting from 1 or 2 d after lambing. The conclusions from Tables 2 and 3 are the same, i.e., LA breeding results in milk with a higher (*P* < 0.05) percentage of fat and protein, but the yield of milk, fat and protein is similar between the two breeds, and EF breeding results in more (*P* < 0.05) lambs born per ewe lambing than does LA breeding.

**Table 3:** **Predicted performance of pure East Friesian and Lacaune 3**-**year**-**old ewes from performance records of crossbred ewes of various percentages of East Friesian and**/**or Lacaune breeding**^**1**,**2**^

	**Breed**	
**Trait**	**East Friesian**	**Lacaune**
Lactation length, d	188.6^a^	180.3^a^
Milk yield, kg	359.3^a^	345.1^a^
Fat yield, kg	20.9^a^	22.1^a^
Fat,%	6.3^a^	6.5^b^
Protein yield, kg	18.0^a^	18.2^a^
Protein,%	5.2^a^	5.3^b^
Litter size, no.	1.97^a^	1.84^b^

^1^Previously unpublished data.

^2^Performance records collected from1996 through 2005 on 1,068 individual ewes with 2,554 lactation records.

^a,b^Means within a row with no superscript in common are different (*P* < 0.05).

Since there have been no new importations of LA genetics and only limited new importations of EF genetics into the U.S. since the late 1990’s and no national or regional programs for genetic improvement of dairy sheep in the U.S., the comparative performance of EF and LA genetics obtained from these studies are expected to be accurate predictions of breed differences that will be observed by commercial dairy sheep producers in the U.S. However, our estimates of breed differences may not be accurate for the current world population of EF and LA sheep. First, our samples of rams from each of the breeds, especially from the LA breed, were small and may not have been good representations of the EF and LA breeds found in Europe in the late 1990’s. Second, rates of genetic improvement in these two breeds over the past 15 to 20 yr are probably different. Evidence suggests that the LA breed in France has most likely made greater genetic improvement in lactation traits than has the EF breed in Germany. From 2005 to 2011, an average of 68 flocks and 855 ewes of the EF breed were milk recorded each year in Germany whereas an average of 384 flocks and 172,946 ewes of the LA breed were milk recorded each year over the same period in France [6]. The French LA genetic improvement program is the most sophisticated and effective program among dairy sheep breeds in the world. In 2006, it was estimated that the recorded LA flocks in France had a rate of genetic improvement of +6 liters of milk per ewe per year [7]. While there are no similar estimates of the genetic trend for milk yield in EF sheep in Germany, it is most likely very much lower than for the LA in France due to a less aggressive genetic improvement program for EF compared to LA. Therefore, a sampling of European EF and LA rams today might result in different conclusions than those arrived at from our samples from the late 1990’s.

#### Lamb survival of East Friesian

There are reports in the literature of poor survival of lambs of high percentage EF breeding in France [8] and Greece [9] compared to other breeds with the primary cause of the increased mortality due to respiratory disease. We observed a similar effect in the early years of our work with EF. As we graded-up our non-dairy sheep to higher percentages of EF breeding by top-crossing with EF rams, our lamb mortality increased. Presented in Table 4 is survival of 483 lambs of different proportions of EF breeding born in 1999 (an early year in our grading-up program from non-dairy to dairy sheep) that we reported in 1999 [10] and 2000 [11]. Lambs over 50% EF breeding had significantly lower (*P* < 0.05) survival rates than lambs with 50% or lower EF breeding during all time periods, and lambs of 50% EF breeding had numerically lower, but not significantly lower, survival rates than lambs with less than 50% EF breeding.

**Table 4:** **Least squares means for lamb survival by percentage of East Friesian breeding of the lamb (1999 lamb crop)**

		**Survival rate,%**		
**Lamb’s % EF breeding**	**No. lambs born alive**	**Birth to weaning**	**Weaning to 7/1/99**	**Birth to 7/1/99**
0	56	96.4 ± 3.5^a^	100.0 ± 2.9^a^	96.4 ± 4.2^a^
>0 to <25	146	96.6 ± 2.2^a^	99.3 ± 1.8^a^	95.9 ± 2.6^a^
≥25 to <50	70	97.1 ± 3.1^a^	98.5 ± 2.6^a^	95.7 ± 3.8^a^
50	60	95.0 ± 3.4^a^	93.0 ± 2.8^a,b^	88.3 ± 4.1^a^
>50	151	83.4 ± 2.1^b^	86.5 ± 1.9^b^	72.2 ± 2.6^b^

^a,b^Within a column, means with a different superscript are different (*P* < .05).

However, a more detailed analysis of lamb survival in this flock for 7,990 lambs born from 1996 through 2011 was reported in an abstract in 2012 [12]. The analysis determined direct and maternal breed effects, individual and maternal heterosis effects, and the effects of the non-genetic factors of lamb type of birth, sex of lamb, month of birth, and age of dam on lamb survival over several age intervals from birth up to 120 d of age. The EF maternal breed effect was significantly negative for lamb survival whereas the EF direct breed effect was generally not significantly different from zero. These results suggest that it is the EF breeding of the ewe and not the EF breeding of the lamb that is responsible for the increased lamb mortality reported in EF breeding programs. It is obvious that more studies are needed to determine the biology of lamb survival in EF populations.

### Weaning systems

In 1998, 99 EF-cross ewes in second and third parity were utilized to compare milk production and lamb growth under three weaning systems [13]. One group of ewes (DY1, n = 31) was weaned from their lambs between 24 and 36 h postpartum and machine milked twice daily for the entire lactation. Their lambs were raised on milk replacer until approximately 30 d of age. Another group of ewes (MIX, n = 35) were separated from their lambs at 1700 h. each day and milked once daily each morning at 0600 h from 24 to 36 h after parturition. After the morning milking, ewes were returned to their lambs. MIX ewes were milked twice daily following permanent weaning of their lambs at approximately 30 d of age. The final group of ewes (DY30, n = 33) were left to raise their lambs and not initially milked. Approximately 30 d postpartum, ewes were weaned from their lambs and milked twice daily for the remainder of their lactation. Milk yields of ewes on the three systems were milk obtained during machine milking only and did not estimate milk consumed by the lambs.

Milk yield differed (*P* < 0.05) among the three weaning systems (DY1 = 260 kg, MIX = 236 kg, and DY30 = 172 kg) (Table 5). Lamb weights at 30 d of age when they were weaned from milk replacer or their dams were not significantly different among the weaning treatment groups (averaged 15 kg across treatments). However, lamb weights at 120 d of age tended to be lighter (*P* < 0.10) for lambs from DY1 ewes (44 kg) than for lambs from DY30 ewes (47 kg). Lambs from MIX ewes had intermediate 120 d weights (46 kg). The lamb growth data suggest that artificial rearing of lambs has a slight negative effect on lamb postweaning gain relative to the effects of either limited suckling or *ad libitum* suckling preweaning. Relative to the DY30 system, income from milk and lamb over additional expenses was + $30.66 per ewe for the MIX system and + $5.05 per ewe for the DY1 system. Of course, these economic returns depend on the value of milk and lambs and their relative values, which do change over time. Any one of the three weaning systems could be the most profitable under different sets of prices for milk and lambs.

**Table 5:** **Ewe lactation**, **lamb growth**, **and economics of three weaning systems**^**1**^

	**Weaning system**		
**Trait**	**DY1**	**MIX**	**DY30**
Ewe lactation traits (n):	(31)	(35)	(33)
Lactation length, d	183.4 ± 5.4	179.2 ± 5.1	182.9 ± 5.5
Machine milking period, d	182.4 ± 5.4^a^	178.2 ± 5.1^a^	152.3 ± 5.5^b^
Commercial milk yield, kg	260.1 ± 9.7^a^	235.8 ± 9.1^b^	171.7 ± 9.9^c^
Fat yield, kg	13.2 ± 0.6^a^	10.9 ± 0.5^b^	8.4 ± 0.6^c^
Fat,%	5.1 ± 0.1^a^	4.5 ± 0.1^b^	4.8 ± 0.1^a,b^
30-d fat,%	4.8 ± 0.2^a^	2.8 ± 0.2^b^	-
Protein yield, kg	13.7 ± 0.5	12.1 ± 0.5	9.0 ± 0.5
Protein,%	5.3 ± 0.1	5.1 ± 0.1	5.2 ± 0.1
Lamb growth traits (n at 120 d):	(64)	(71)	(73)
30-d weight, kg	15.4 ± 0.4	14.5 ± 0.6	15.0 ± 0.5
120-d weight, kg	43.7 ± 1.2^f^	45.9 ± 1.8^e^	47.3 ± 1.6^d^
Economics:			
Total lamb & milk receipts^2^, $	506.52 ± 18.07^a^	458.23 ± 17.05^b^	415.25 ± 18.53^b^
Additional expenses^3^, $	87.16 ± 2.98^a^	14.40 ± 3.04^b^	-
Receipts – additional expenses, $	420.86 ± 16.87	446.47 ± 15.91	415.81 ± 17.30

^1^Study conducted in 1998.

^2^Total lamb weight at 120 d of age per ewe and commercial milk yield per ewe were valued at $1.87 and $1.32/kg, respectively.

^3^Additional labor and supply expenses per ewe relative to the DY30 system during the first 30 d of lactation.

^a,b,c^Means within a row with no superscript in common are different (*P* < 0.05).

^d,e,f^Means within a row with no superscript in common are different (*P* < 0.10).

The economic returns assumed no price differentials for milk composition. Milk from MIX ewes had a lower (*P* < 0.05) fat percentage (4.53%) than milk from either DY1 (5.06%) or DY30 (4.81%) ewes. The lower milk fat percentage of MIX ewes was most dramatic during the first 30 d when they were suckling their lambs during the day. During this period, MIX ewes had a milk fat percentage of 2.80% while DY1 ewes at the same stage of lactation had a milk fat percentage of 4.82%. If price discounts were in place for low-fat milk, the MIX system would have less of an economic advantage than projected in this study. Even so, the MIX system is attractive over the other two systems because the ewes raise their lambs and still produce 85% as much milk as DY1 ewes.

Subsequently, a more detailed study conducted on the UW-Madison campus determined that the low milk fat from MIX ewes while they are nursing their lambs is due to failure of milk ejection from the udder alveoli due to failure of oxytocin release in these ewes during milking [14]. Oxytocin is released as a result of teat and udder stimulation, usually at the time of suckling. Oxytocin is an integral part of milk ejection (the contraction of the alveoli within the udder that causes secreted milk to flow down a system of ducts and canals into the storage part of the udder known as the cistern). During machine milking, if there is no release of oxytocin, secreted milk remains in the alveoli along with large quantities of milk fat. The MIX ewes experienced impairment of oxytocin release and the milk ejection reflex because they knew that after milking they would be reunited with their lambs. The milking machine captured their cisternal milk but not their alveolar milk where most of the fat is found.

### Fat supplementation

Previous studies with ewes had shown an increased milk fat content when ewes were fed calcium salts of fatty acids (CSFA) during lactation [15-18]. Megalac Rumen Bypass Fat (Church and Dwight Co., Inc.), a CSFA, was added to the diets of EF-cross dairy ewes in early lactation in a study we conducted in 1999 to determine if the treatment could counteract the negative effects of the MIX weaning system on milk fat content [19]. The CSFA was mixed in a diet of whole shelled corn and a protein pellet and fed in the milking parlor to provide 0.10 kg of CSFA per ewe per day.

Ewes (n = 274) lambed over a 6-wk period starting on February 10, 1999 and were randomly allocated to a DY1 or MIX system as they lambed. The CSFA supplementation started on March 3, 1999 and ran for 8 wk. During the first and third 2-wk periods, all ewes received the unsupplemented diet, and during the second and fourth 2-wk periods, all ewes received the CSFA supplemented diet.

The CSFA supplementation had no effect on milk yield but tended to depress milk protein percentage in both DY1 and MIX ewes. The CSFA supplementation resulted in a large increase (*P* < 0.05) in milk fat percentage of approximately +1.19 percentage units in DY1 ewes but had no effect on milk fat percentage of MIX ewes during the milking-suckling period. Therefore, fat supplementation may be a method to increase fat percentage in ewes that have weaned their lambs, but it is not a solution to the low fat percentage of milk from ewes that are still suckling their lambs. Milk fat synthesis was probably not impaired in CSFA-supplemented MIX ewes, but the milk fat was retained within the udder until it was removed by the suckling lamb.

### Milking intervals

#### Three-times-a-day milking

During 2000, 125 multiparous EF crossbred ewes were utilized to compare traditional twice-a-d milking (2×, n = 72) with three-times-a-d milking (3×, n = 53) during the first 30 d of lactation [20]. The 2× ewes were milked at 0630h and 1730h, and the 3× ewes were milked at 0600h, noon, and 1800h each day. After d 30 of lactation, all ewes were milked twice-a-d. All lambs were weaned from their dams within 24 h after parturition, and ewes were immediately assigned to a milking treatment. During the 30-d treatment period, 3× ewes produced a total of 12.6 kg more (+15.2%, *P* < 0.05) milk than 2× ewes (95.2 versus 82.6 kg, respectively). During the 30-day treatment period, the 3× ewes had increased net income of $10.00 per ewe over the 2× ewes. There were no carry-over effects of the treatments in later lactation with 3× and 2× ewes having the same milk yield in wk 7 after both groups had been on 2-times-a-d milking starting in wk 5.

#### 16-Hour milking interval

A trial was conducted in 2001 to determine if the milking interval could be extended from 12 to 16 h starting in mid-lactation without a significant drop in milk yield [21]. Forty-eight third lactation EF crossbred ewes were utilized. Twenty-four ewes were kept on the 12 h milking interval (12H, milked daily at 0600h and 1800h) and 24 ewes were switched from the 12H interval on approximately d 90 of lactation to a 16 h milking interval (16H, milked at 0600h and 2200h one day and at 1400h the following day and then repeating). Lactation performance was measured through d 180 of lactation.

During the 90-d treatment period, 16H ewes produced about 28% more (*P* < 0.05) milk at each 0600h milking than 12H ewes, but there was no difference between treatments in the total amount of milk produced during the entire treatment period (Table 6). The percentage of fat and protein and somatic cell count were not different between the two treatments. From mid- to late lactation, it appears that the number of milkings can be reduced by 25% without a decrease in milk production. This is possible because it has been shown that a larger proportion of the milk yield of dairy goats and dairy sheep is cisternal milk [22] compared to the milk yield of cows [23], i.e, small dairy ruminants have a greater capacity to store milk between milkings than do cows.

**Table 6:** **Lactation performance of ewes**^**a **^**milked at 12 or 16 hour intervals from day 90 to 180 of lactation**

	**Milking interval**	
**Trait**	**12 hour**	**16 hour**
Total number of milkings	180	135
6 a.m. milk yield, kg	0.65 ± 0.03^c^	0.83 ± 0.03^b^
Average 24-hour milk yield, kg	1.34 ± 0.06	1.35 ± 0.06
Total milk yield, kg	119.1 ± 5.3	118.0 ± 5.3
Total parlor time for 24 ewes, h	38.1	27.9

^a^Twenty-four third lactation East Friesian crossbred ewes were used on each treatment.

^b,c^Means within a row with no superscript in common are different (*P* < 0.05).

No test of statistical significance was possible for total number of milkings or total parlor time.

### Machine stripping

Due to the large cisternal storage capacity and non-vertical teat placement in most dairy ewes, machine stripping is commonly performed to remove milk not obtained by the machine. However, stripping requires individual manual intervention, lengthens the milking routine, and could inadvertently lead to overmilking of other ewes in the parlor [24]. A study was conducted in 2000 to estimate the effect of omission of machine stripping on milk production and parlor throughput of dairy ewes [25].

Forty-eight multiparous ewes of 50 to 75% EF breeding that had been machine milked and stripped twice daily from d 0 to 79 post-partum, were randomly assigned to two stripping treatments for the remainder of lactation: normal stripping (S, n = 24), or no stripping (NS, n = 24). Ewes were milked in a 2 × 12 high-line Casse system milking parlor at 0600h and 1800h each d.

Ewes that were not stripped yielded 14% less (*P* < 0.05) commercial milk during the experiment (NS = 122.7 kg, S = 105.6 kg), but had a similar lactation length (104 d), milk composition, and somatic cell count compared to S ewes. Average machine-on time for S ewes was 10.4 seconds per ewe longer (*P* < 0.10) than for NS ewes because of stripping, which may have resulted in overmilking of some ewes in the S group.

A milking simulation in a double-12 parlor with one or two milkers and stripping or no stripping was conducted. With one milker, elimination of stripping increased the number of ewes milked per hour by 49% (from 103 to 153 ewes per h), and the number of ewes overmilked per side decreased from 11 out of 12 to 0 out of 12. With two milkers, elimination of stripping increased the number of ewes milked per hour by 20% (from 138 to 166 ewes per h), and the number of ewes overmilked per side decreased from 4 out of 12 to 0 out of 12.

These results collectively indicate that elimination of machine stripping will reduce milk yield per ewe, but the loss in milk yield may be somewhat or completely compensated for by increased parlor throughput and the udder health advantages resulting from not overmilking ewes.

### Effect of number of corpora lutea on milk yield

The effects of corpora lutea on milk production were examined in 24 second lactation EF crossbred ewes in a study conducted in 1999 [26]. Ewes were synchronized using intravaginal progesterone (controlled intravaginal drug-releasing (CIDR®, Zoetis United States) device), PGF2*α*, and gonadotropins. After ovulation, corpora lutea (CL) were counted via laparoscopy on d 4 and 11. On d 5, ewes received either saline (CLYES, n = 12) or PGF2*α* (CLNO, n = 12) to allow CL persistence (2.4 ± 0.3 CL on d 11) or regression (0 CL on d 11), respectively. Each ewe received two CIDR from d 5 to 18 to maintain high concentrations of plasma progesterone (P4) and to suppress estradiol (E2). Each ewe received PGF2*α* on d 18 to regress all CL. Data were collected during three periods (pre-treatment: d 0 to 5; treatment: d 6 to 18; post-treatment: d 19 to 25). Milk yield and milking time were recorded daily, milk samples were obtained for analyses of fat and protein, and blood samples were collected for P4 and E2 immunoassay.

During treatment, CLYES ewes had higher (*P* < 0.05) milk yield (1.56 vs. 1.44 kg/d), milk fat (92.2 vs. 81.1 g/d), and milk protein (83.7 vs. 77.5 g/d) compared with CLNO ewes, respectively. Differences were maintained post-treatment, despite luteolysis in CLYES ewes. Serum P4 concentrations were greater (*P* < 0.05) during the treatment period for CLYES compared to CLNO ewes (5.3 vs. 2.9 ng/mL, respectively). Estradiol concentrations did not differ between treatments and were low after d 5.

While this study showed an increase in milk yield from ewes with active CL, the biological mechanism was not definitively determined. The increase in milk yield was not due to estradiol, but it may have been a direct effect of the increased P4 concentrations in the CLYES ewes. However, P4 concentrations were not related to milk yield in an older study [27]. While oxytocin levels were not measured in this study, others have reported higher oxytocin levels in ewes with CL compared to ewes without CL [28], and the higher levels of oxytocin may result in greater transfer of milk from the alveoli to the cistern between milkings in CLYES ewes compared to CLNO, resulting in their greater milk yield.

### Prepartum photoperiod and milk production

Previous studies have shown that long photoperiods during established lactation increase milk production in dairy cattle [29] and dairy sheep [30], and later studies in dairy cattle [31] and dairy goats [32] suggested that short photoperiods prepartum increased milk yield in the subsequent lactation. The proposed mechanism of function was the level and role of circulating prolactin in mammary development. We conducted a study in 2005-2006 to evaluate the effect of prepartum photoperiod on milk production, milk composition, and prolactin concentration of dairy ewes [33].

Twenty two multiparous crossbred dairy ewes of EF and LA breeding were exposed to short day prepartum photoperiod (SDPP; 8 h of light: 16 h of dark) or long day prepartum photoperiod (LDPP; 16 h of light: 8 h of dark) starting on December 5, 2005 for at least 6 wk prepartum in adjacent environmentally-controlled rooms. Blood samples were collected from each ewe twice weekly during the prepartum period, at the time of lambing, and 1 wk after lambing and analyzed for prolactin concentrations. After lambing, lambs were removed and raised on milk replacer, and all ewes were relocated to a common room in the same facility with 12 h of light: 12 h of dark and milked twice per day in this facility for 53 ± 3 d (trial period). On April 6, 2006 all ewes were returned to the main flock of dairy ewes and maintained under ambient light (post-trial period) under commercial management conditions. The total milking period (trial period + post-trial period) was 180 ± 6 d. Table 7 presents the results from the trial.

**Table 7:** Average daily milk production and milk composition of multiparous crossbred dairy ewes exposed to a short or long photoperiod during the prepartum period

	**Trial period (53 d)**			**Trial** **+** **post**-**trial period (180 d)**		
**Trait**	**SDPP**^**1**^	**LDPP**^**2**^	***P***	**SDPP**^**1**^	**LDPP**^**2**^	***P***
Milk, kg/d	2.43 ± 0.051	2.29 ± 0.051	0.053	1.76 ± 0.051	1.60 ± 0.050	0.031
Fat,%	6.04 ± 0.102	5.51 ± 0.104	0.000	6.28 ± 0.110	6.48 ± 0.107	0.216
Protein,%	4.61 ± 0.057	4.54 ± 0.057	0.448	5.23 ± 0.064	5.12 ± 0.062	0.241

^1^Short day prepartum photoperiod (8 h light: 16 h dark), n = 11.

^2^Long day prepartum photoperiod (16 h light: 8 h dark), n = 11.

During the first 8 wk of lactation, SDPP ewes tended to produce more (*P* = 0.053) milk than LDPP ewes (2.43 vs. 2.29 kg/d, respectively), and the milk of SDPP ewes had a greater (*P* < 0.01) fat percentage than that of LDPP ewes (6.04 vs. 5.51%, respectively). For the entire lactation period of 180 d, SDPP ewes produced more (*P* < 0.05) test day milk than LDPP ewes (1.76 vs. 1.60 ± 0.05 kg/d, respectively), but there were no differences in milk fat or protein percentages.

**Figure 1:**
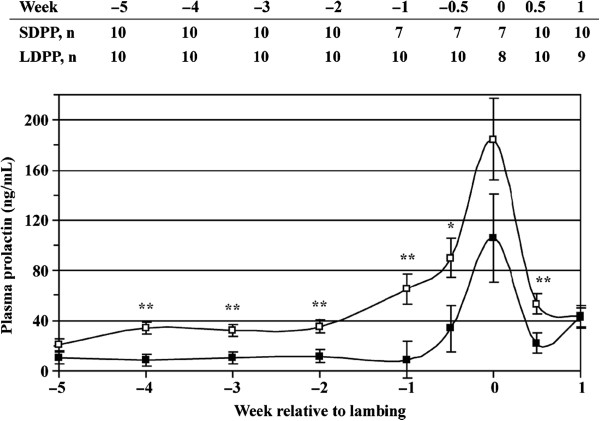
**Mean concentration of plasma prolactin of ewes exposed to short day photoperiod (SDPP; 8 h of light: 16 h of dark) or long day prepartum photoperiod (LDPP; 16 h of light: 8 h of dark).** The table presents the number of ewes per treatment (n) on each week relative to lambing. Error bars indicate SEM. *Least squares means within a test day are different (*P* < 0.05). **Least squares means within a test day are different (*P* < 0.01).

Ewes in both treatments experienced a prolactin (PRL) surge at lambing (Figure 1), but SDPP ewes had lower (*P* < 0.05) circulating prolactin concentration than LDPP ewes from 4 to 0.5 wk before lambing (14.7 vs. 51.3 ± 4.2 mg/dL, respectively). Similar results have been reported in dairy cows and dairy goats, and some of these studies have conducted more extensive hormonal studies. A possible cascade of events to describe the effect of prepartum photoperiod on milk production may be as follows: 1) short photoperiod in prepartum ruminants results in decreased circulating PRL concentrations; 2) decreased PRL stimulates the increased expression of mRNA for PRL receptors, resulting in a greater number of PRL receptors on mammary secretory epithelial cells; and 3) the natural increase in circulating PRL at parturition stimulates more extensive differentiation and commitment of mammary secretory epithelial cells to produce lactose, thereby increasing milk production in SDPP-treated animals.

These data suggest that decreased prepartum photoperiod may be important for increasing milk production in dairy ewes and may provide a management strategy for dairy sheep producers to increase milk yield. Ewes in late gestation in early winter when day length is short may be expected to produce more milk than ewes in late gestation in late winter or spring when daylength is increasing.

### Grazing and supplementation on pasture

#### Pasture compared to drylot for lactating ewes

In 1998, 97 EF crossbred ewes had been maintained in drylot from early to mid-lactation where they received grain twice per day in the parlor at milking and alfalfa hay during the d in drylot. From mid-lactation to the end of lactation, 48 ewes remained in the drylot and the remaining 49 ewes were grazed during the day on a kura clover-orchard grass pasture. The pastured ewes had 10.5% greater (*P* < 0.05) lactation milk yields than the ewes in drylot (184 versus 167 kg) (unpublished data). As a result of this trial, grazing lactating ewes during the grass-growing season has become a general management practice at the Spooner Agricultural Research Station.

#### Supplementation on pasture

Trials were conducted in 2005 and 2006 to determine the efficacy of supplementation of lactating ewes while grazing high quality kura clover-orchard grass pastures [34].

In trial 1 conducted in 2005, 56 three-yr-old grazing crossbred dairy ewes of EF and LA breeding in early (21 d in milk, n = 10) or late (136 d in milk, n = 46) lactation were fed 0 (n = 28) or 0.82 (n = 28) kg DM/d per ewe of supplement (16.5% crude protein mixture of corn and a soybean meal-based high-protein pellet) in a 2 × 2 factorial arrangement of treatments. Individual ewe test d milk yield was collected every 14 d for 84 d of the grazing season. Supplementation had similar effects in both early and late lactation ewes. Supplemented ewes had higher (*P* < 0.01) milk production (1.59 vs. 1.36 kg/d, respectively), lower (*P* < 0.10) milk fat percentage (5.75 vs. 6.00%, respectively), lower (*P* < 0.01) milk protein percentage (4.84 vs. 5.04%, respectively) but more (*P* < 0.01) fat and protein corrected milk production (1.09 vs. 0.95 kg/d, respectively) than unsupplemented ewes (Table 8).

**Table 8:** **Lactation performance of supplemented**^**1 **^**or unsupplemented crossbred dairy ewes**^**2 **^**grazing a legume**-**grass pasture**

**Trait**	**Unsupplemented**	**Supplemented**
Test day milk yield, kg	1.36 ± 0.04^b^	1.59 ± 0.04^a^
Test day FPCM^3^ yield, kg	0.95 ± 0.04^b^	1.09 ± 0.04^a^
Milk fat,%	6.00 ± 0.09^c^	5.75 ± 0.09^d^
Milk protein,%	5.04 ± 0.04^a^	4.84 ± 0.04^b^
Milk urea nitrogen, mg/dL	24.9 ± 1.58	25.1 ± 1.51

^1^0.82 kg DM/d of a 16.5% crude protein mixture of corn and a soybean meal-based high-protein pellet.

^2^Multiparous crossbred ewes of East Friesian and Lacaune breeding, n = 28 ewes per treatment.

^3^6.5% fat-corrected and 5.8% protein-corrected milk yield.

^a,b^Means within a row with no superscript in common are different (*P* < 0.01).

^c,d^Means within a row with no superscript in common are different (*P* < 0.10).

Protein has a high nitrogen content. If protein intake is in excess of the needs of the rumen microflora, high levels of protein nitrogen in the form of urea are excreted in the urine, feces, and milk. Therefore, milk urea nitrogen (MUN) is a good indicator of the efficiency of protein utilization by the microflora of the rumen. MUN levels were similar between supplemented and unsupplemented ewes (average of 25.0 mg/dL) but were above recommended levels for dairy ewes (14 to 22 mg/dL, [35]), indicating excess protein (N) or insufficient energy intake. It appeared that the ewes benefited from the increased energy in the supplement. However, the protein in the pasture, which varied from 16 to 30% during the grazing season, may have been adequate for the level of milk production of these ewes, and the additional protein in the 16.5% crude protein supplement may not have been necessary. This observation resulted in a second supplementation trial the following grazing season.

In trial 2 in 2006, 96 two-, three-, and four-year-old grazing crossbred dairy ewes of EF and LA breeding in mid-lactation (112 d in milk) were randomly assigned to 4 treatments of 0.00, 0.41, 0.82, or 1.24 kg DM/d per ewe of whole shelled corn to determine if a high energy supplement, but of lower protein content than the supplement used in trial 1, may provide a better complement to the high protein content of the pasture [34]. There was a linear increase (*P* < 0.01) in test-day milk yield and test day fat- and protein-corrected milk yield, a linear decrease (*P* < 0.01) in milk fat percentage, and no significant change in milk protein percentage with increasing amounts of corn supplementation (Table 9). Even though the quadratic effects of supplementation levels on milk yields were not statistically significant, the supplementation means indicate that there was no advantage of supplementation above 0.82 kg DM/ewe/d. MUN levels for all four groups were within the range suggested for dairy sheep and decreased (*P* < 0.001) with increasing amounts of corn supplementation. This suggested that protein levels in the high quality legume-grass pasture were adequate for milk production in these ewes and utilization of pasture protein improved with increasing dietary energy intake from whole shelled corn.

**Table 9:** **Lactation performance of crossbred dairy ewes**^**1 **^**unsupplemented or supplemented with corn during the grazing season**

**Trait**	**Whole shelled corn supplementation, kg DM/ewe/d**				**SEM**	**Linear contrast, *P***-**value**
	**0.00**	**0.41**	**0.82**	**1.24**		
Test day milk yield, kg	1.30^a^	1.32^a^	1.41^b^	1.44^b^	0.03	0.001
Test day FPCM^2^ yield, kg	1.26^ab^	1.25^a^	1.35^c^	1.33^bc^	0.03	0.006
Milk fat,%	6.26^b^	6.40^b^	6.09^b^	5.89^a^	0.11	0.001
Milk protein,%	5.29	5.41	5.37	5.39	0.04	0.093
Milk urea nitrogen, mg/dL	18.9^a^	17.1^b^	13.6^c^	13.6^c^	0.3	0.001

^1^Multiparous crossbred ewes of East Friesian and Lacaune breeding, n = 24 ewes per treatment.

^2^6.5% fat-corrected and 5.8% protein-corrected milk yield.

^a,b,c^Means within a row with no superscript in common are different (*P* < 0.05).

### Level of protein and rumen undegraded (by-pass) protein

Dietary protein is provided to ruminants in the form of rumen degraded protein (RDP) and rumen undegraded (by-pass) protein (RUP). RDP is utilized by the microflora of the rumen, and the microbial protein is then utilized by the animal. RDP fed in excess of the needs of the rumen microflora is excreted in the feces or as urea in the urine or milk. Rumen undegraded protein cannot be utilized by the rumen microflora but is utilized directly by the animal. High-producing ruminants, like lactating dairy ewes, may increase their productivity if RUP is added to rations already adequate in RDP. A study was conducted in 2008 to test this hypothesis [36].

Three diets were formulated to provide similar energy concentrations and varying concentrations of RDP and RUP: 12% RDP and 4% RUP (12-4) included basal levels of RDP and RUP, 12% RDP and 6% RUP (12-6) included additional RUP, and 14% RDP and 4% RUP (14-4) included additional RDP. Diets were composed of alfalfa-timothy cubes, whole and ground corn, whole oats, dehulled soybean meal, and expeller soybean meal (SoyPlus, West Central, Ralston, IA). Eighteen third lactation crossbred dairy ewes of EF and LA breeding in mid-lactation were divided by milk yield (low and high) into 2 blocks of 9 ewes each and were randomly assigned within block (low and high) to 3 pens of 3 ewes each. Dietary treatments were arranged in a 3 × 3 Latin square within each block and applied to pens for 14-d periods. Milk yield and composition was determined during the last 4 d of each treatment period.

There was no effect of dietary treatment on dry matter intake. The 18% crude protein diet with the high level of RDP (14-4) resulted in no more milk production than obtained with the 16% crude protein diet with a lower level of RDP and the same level of RUP (12-4). However, the 18% crude protein diet with the high level of RUP (12-6) increased (*P* < 0.01) milk yield over both the 14-4 and 12-4 diets (Table 10). This is strong evidence for the inclusion of RUP in diets of lactating ewes.

**Table 10:** **Lactation performance of dairy ewes**^**1 **^**fed diets with varying levels of rumen degraded** (**RDP**) **and undegraded** (**RUP**) **protein**

	**% RDP:%RUP**			
**Trait**	**12:6**	**14:4**	**12:4**	**SEM**
Test day milk yield, kg	2.05^a^	1.80^b^	1.79^b^	0.07
Milk fat,%	6.13	6.37	6.18	0.25
Milk protein,%	4.74	4.95	4.80	0.14
Milk urea nitrogen, mg/dL	26.3^a,b^	27.4^a^	23.4^b^	1.4

^1^Third lactation crossbred ewes of East Friesian and Lacaune breeding, n = 6 pens of 3 ewes each per treatment in a Latin square design.

^a,b^Means within a row with no superscript in common are different (*P* < 0.05).

Milk urea N concentration was greater (*P* < 0.05) in the 14–4 diet and tended to be greater (*P* < 0.10) in the 12–6 diet compared with the 12–4 diet, indicating that the excretion of urea N in this study was more closely related to dietary crude protein concentration than to protein degradability (Table 10).

### Legume content of forage

Our previous trials with dairy ewes fed stored feeds indicated a positive effect of RUP supplementation on milk yield. However, dairy sheep production in the United States is primarily based on grazing mixed grass-legume pastures, which contain a high proportion of RDP. Two trials were conducted in 2008 and 2009 to evaluate the effects of RUP protein supplementation and varying levels of the legume, alfalfa, in legume-grass forages on lactation performance [37].

In a cut-and-carry trial conducted in 2008, 16 multiparous crossbred dairy ewes of EF and LA breeding in mid-lactation were randomly assigned one of two protein supplementation treatments, receiving either 0.0 or 300 g of a high-RUP protein supplement (Soy Pass, LignoTech USA Inc., Rothschild, WI) per d. Within supplementation treatment, ewes were full-fed freshly cut forage of varying percentages of alfalfa:orchardgrass dry matter: 0:100, 25:75, 50:50, or 75:25. Supplementation with a high-RUP source tended to increase (*P* < 0.10) milk yield by 9%. Milk yield (*P* < 0.10), milk protein yield (*P* < 0.05), and MUN (*P* < 0.05) increased with increased percentage of alfalfa (Table 11).

**Table 11:** **Lactation performance of dairy ewes**^**1 **^**supplemented with rumen undegraded protein** (**RUP**) **and fed or grazing forage of varying proportions of alfalfa**

**Trait**	**RUP Supplement**			**% alfalfa in forage**				
	**No**	**Yes**	**SEM**	**0**	**25**	**50**	**75**	**SEM**
Cut-and-carry trial:^2^								
Milk yield, kg/d	1.79^f^	1.95^e^	0.06	1.74^g^	1.85^f^	1.94^e^	1.95^e^	0.06
Fat yield, g/d	122	123	3	117	122	124	127	4
Protein yield, g/d	90	95	2	85^b^	90^b^	96^a^	98^a^	3
Milk urea nitrogen, mg/dL	12.3^b^	15.1^a^	0.8	10.9^d^	12.7^c^	14.3^b^	16.8^a^	0.7
Grazing trial:^3^								
Milk yield, kg/d	1.65	1.82	0.16	1.55^g^	1.78^f^	1.87^e^		0.11
Fat yield, g/d	105	115	11	102	113	116		8
Protein yield, g/d	84	94	7	78^a^	90^a,b^	98^b^		5
Milk urea nitrogen, mg/dL	18.2	19.8	1.0	15.0^b^	19.8^a^	22.1^a^		0.8

^1^Multiparous crossbred ewes of East Friesian and Lacaune breeding.

^2^n = 16 ewes; 8 pens of 2 ewes each. 4 pens randomly assigned to each supplement treatment. Within supplementation treatment, pens were randomly assigned to 1 of 4 forage treatments, which were applied in a 4 × 4 Latin square design for 10-d periods.

^3^n = 12 ewes; 3 groups of 4 ewes each. 2 ewes in each group randomly assigned to each supplement treatment. Grazing treatments were arranged in a 3 × 3 Latin square design and randomly applied to groups for 10-d periods.

^a,b,c,d^Means within a row and treatment with no superscript in common are different (*P* < 0.05).

^e,f,g^Means within a row and treatment with no superscript in common are different (*P* < 0.10).

In a grazing trial conducted in 2009, 12 multiparous crossbred dairy ewes of EF and LA breeding in mid-lactation were randomly assigned to receive either 0.0 or 300 g of a high-RUP protein supplement (SoyPlus, West Central Cooperative, Ralston, IA) per day. Within supplementation treatments, ewes grazed paddocks that contained the following percentages of surface area of pure stands of alfalfa:orchardgrass: 0:100, 25:75, or 50:50. Milk yield (*P* < 0.10), milk protein yield (*P* < 0.05), and MUN (*P* < 0.05) increased with increased percentage of alfalfa in the paddock (Table 11).

In conclusion, supplementing with high-RUP protein tended to increase milk yield, and increasing the proportion of alfalfa in the diet increased dry matter intake, milk yield, and protein yield of lactating dairy ewes fed or grazing fresh forage.

## Conclusions

The Spooner Agricultural Research Station of the University of Wisconsin-Madison has made significant research contributions to the dairy sheep industry of North America since its establishment in 1993 in several areas; especially in new breed introduction and evaluation, lactational physiology, nutrition of the lactating ewe, and general dairy sheep management. Both the EF and LA dairy breeds were introduced to the industry and evaluated by the Spooner Station. Both breeds produced significantly more milk than traditional meat-wool breeds found in the U.S. East Friesian ewes produced more lambs and slightly more milk than LA ewes whereas LA ewes produced milk with a higher percentage of fat and protein than EF ewes. Studies in lactational physiology showed that ewes with active corpora lutea had increased milk yields, oxytocin release during milking was required to obtain normal fat percentages in milk, large udder cisterns of dairy ewes allowed for increased milking intervals, and short daylengths during late pregnancy resulted in increased milk yield. In the nutrition area, legume-grass pastures and forages with a higher percentage of legumes resulted in increased milk production. Grazing ewes responded to additional supplementation with increased milk yield, but it was important to match the supplement to the quality of the grazing. Ewes on high quality legume-grass pastures that are high in rumen degradable protein responded with increased milk production to supplements high in energy and/or high in rumen undegraded protein.

## Competing interest

The authors declare that they have no competing interests.

## Authors’ contribution

All experiments were conducted under the direction of DLT and YMB. Specific experiments were designed and conducted by either BCM or CMM as part of their M.S. and Ph.D programs, and these experiments can be identified by BCM or CMM being the first author of the reference to the experiment. All authors read and approved the final manuscript.
